# Microvesicles Derived from Inflammation-Challenged Endothelial Cells Modulate Vascular Smooth Muscle Cell Functions

**DOI:** 10.3389/fphys.2016.00692

**Published:** 2017-01-12

**Authors:** Qunwen Pan, Hua Liu, Chunyan Zheng, Yuhui Zhao, Xiaorong Liao, Yan Wang, Yanfang Chen, Bin Zhao, Eric Lazartigues, Yi Yang, Xiaotang Ma

**Affiliations:** ^1^Guangdong Key Laboratory of Age-Related Cardiac and Cerebral Diseases, Institute of Neurology, Affiliated Hospital of Guangdong Medical UniversityZhanjiang, China; ^2^College of Health Science, Wuhan Sports UniversityWuhan, China; ^3^Department of Neurology and Stroke Center, The First Affiliated Hospital, Sun Yat-Sen UniversityGuangzhou, China; ^4^Department of Pharmacology and Toxicology, Boonshoft School of Medicine, Wright State UniversityDayton, OH, USA; ^5^Department of Pharmacology and Experimental Therapeutics, Louisiana State University Health SciencesNew Orleans, LA, USA

**Keywords:** brain endothelial cells, microvesicles, brain vascular smooth muscle cells, cell function, inflammation

## Abstract

**Purpose:** Microvesicles (MV) can modulate the function of recipient cells by transferring their contents. Our previous study highlighted that MV released from tumor necrosis factor-α (TNF-α) plus serum deprivation (SD)-stimulated endothelial progenitor cells, induce detrimental effects on endothelial cells. In this study, we investigated the potential effects of endothelial MV (EMV) on proliferation, migration, and apoptosis of human brain vascular smooth cells (HBVSMC).

**Methods:** EMV were prepared from human brain microvascular endothelial cells (HBMEC) cultured in a TNF-α plus SD medium. RNase-EMV were made by treating EMV with RNase A for RNA depletion. The proliferation, apoptosis and migration abilities of HBVSMC were determined after co-culture with EMV or RNase-EMV. The Mek1/2 inhibitor, PD0325901, was used for pathway analysis. Western blot was used for analyzing the proteins of Mek1/2, Erk1/2, phosphorylation Erk1/2, activated caspase-3 and Bcl-2. The level of miR-146a-5p was measured by qRT-PCR.

**Results:** (1) EMV significantly promoted the proliferation and migration of HBVSMC. The effects were accompanied by an increase in Mek1/2 and p-Erk1/2, which could be abolished by PD0325901; (2) EMV decreased the apoptotic rate of HBVSMC by approximately 35%, which was accompanied by cleaved caspase-3 down-regulation and Bcl-2 up-regulation; (3) EMV increased miR-146a-5p level in HBVSMC by about 2-folds; (4) RNase-treated EMV were less effective than EMV on HBVSMC activities and miR-146a-5p expression.

**Conclusion:** EMV generated under inflammation challenge can modulate HBVSMC function and fate via their carried RNA. This is associated with activation of theMek1/2/Erk1/2 pathway and caspase-3/Bcl-2 regulation, during which miR-146a-5p may play an important role. The data suggest that EMV derived from inflammation-challenged endothelial cells are detrimental to HBVSMC homeostatic functions, highlighting potential novel therapeutic targets for vascular diseases.

## Introduction

Vascular endothelial cells (EC) and smooth muscle cells (VSMC) are the main components of vascular parenchyma and play important roles in vascular homeostasis (Rudijanto, 2007; Chang et al., 2014). Vascular injury and inflammation affect the normal function of VSMC and play a major role in atherogenesis (Gambardella and Santulli, 2016). Various inflammatory cytokines, such as Tumor necrosis factor-α (TNF-α), Interferon-γ (IFN-γ), and Interleukin-2 (IL-2) have been associated with dysfunction in EC and VSMC, which are among the key contributors resulting in various vascular diseases, such as atherosclerosis (AS), hypertension, and vascular stenosis (Ho et al., 2010; Lu et al., 2013; Stone et al., 2013; Zhu et al., 2014). Studies have demonstrated that the pathogenesis of AS is closely associated with the dysregulation of VSMC proliferation, migration, and apoptosis (Shen et al., 2014; Zhu et al., 2014). EC provide an interface between blood and vessel walls, interact in close proximity with VSMC, and contribute to VSMC proliferation and migration (Nagel et al., 1994; Zitman-Gal et al., 2015). However, the underlying mechanisms of EC-VSMC interactions are not fully understood.

Endothelial microvesicles (EMV) are small vesicles 0.1–1 μm in size, which are released when endothelial cells undergo stress, activation or apoptosis (Boulanger et al., 2006; Burger et al., 2013). They harborthe characteristics of their parent cells which make them usefulas potential biomarkers for vascular diseases (Jy et al., 2004; Burger et al., 2013). Moreover, EMV could modulate target cell function through transferring their contents to various recipient cells (Mause and Weber, 2010). Mounting evidence suggests that EMV could regulate EC activation and permeability (Jansen et al., 2013), leukocytes adhesion (Angelot et al., 2009), and platelet activation (Héloire et al., 2003). Of note, the effect of EMV on the recipient cells is dependent on the stimulus under which EMV are released (Jansen et al., 2013; Pan et al., 2016). Among the contents of EMV, miRNA play an important role in the effects of MV on regulating EC and VSMC functions (Tréguer et al., 2012; Jansen et al., 2013). Hergenreider recently reported that miR143/miR145 rich MV from human umbilical vein EC could influence the expression of Ets-like protein 1 (ELK1), Krüppel-like factor 4 (KLF4), and Matrix metalloproteinase3 (MMP3) genes in VSMC (Hergenreider et al., 2012). Moreover, several microRNA have been known to modulate VSMC proliferation and differentiation phenotypes (Cheng et al., 2009; Liu et al., 2009). Among them, miR-146a-5p, an important regulator of inflammation, has been demonstrated to promote VSMC proliferation and migration (Wang et al., 2015). However, the functional roles of EMV released from inflammation-challenged EC on VSMC are unknown, and whether EMV could transfer miR-146-5p to VSMC under this challenged environment is unclear.

TNF-α is an established pro-atherosclerotic factor inducing vascular inflammation injury (Zheng et al., 2013; Zhang et al., 2014). Serum deprivation (SD) is also an important apoptotic stimulus contributing greatly to endothelial dysfunction (Scioli et al., 2014). Therefore, co-culture of EC with TNF-α and SD offers a good model to mimicking ischemia and inflammation in ischemic cardiovascular diseases (Wang et al., 2013). Our previous study has demonstrated that MV released from endothelial progenitor cells under TNF-α and SD environment had detrimental effects on EC function (Wang et al., 2013).

Additionally, the Mek-Erk pathway was reported to be involved in inflammation-induced VSMC proliferation and migration (Lin et al., 2016). Caspase-3 and Bcl-2 are important regulators involved in VSMC apoptosis (Su et al., 2015). Thus, Mek-Erk pathway, Caspase3, and Bcl2 proteins are important factors for the regulation of VSMC functions.

Above all, we hypothesize that EMV derived from HBMEC under inflammatory stimuli could modulate HBVSMC functions via their carried miRNA, and the underlying mechanisms may be associated with the Mek1/2/Erk1/2 and Caspase-3/Bcl-2 Pathways. In this study, we investigated the potential effects of EMV released from human brain microvascular endothelial cells (HBMEC) under TNF-α plus SD stimulation on the proliferation, migration, and apoptosis function of human brain vascular smooth cells (HBVSMC). To determine the role of the EMV-carried RNA, we treated EMV with RNase A and the level of miR-146a-5p in EMV and HBVSMC was assessed. Signaling pathway proteins which are closely associated with proliferation, migration, and apoptosis, such as Mek1/2, Erk1/2, phosphorylated Erk1/2, cleaved caspase-3, and Bcl-2 were examined to explore the underlying mechanisms.

## Materials and methods

### Cell culture

HBMEC were obtained from Shanghai Bioleaf Biotech Co. Ltd. HBVSMC were purchased from Sciencell Research Laboratories, USA. The cells were cultured on 100-mm cell culture dishes in DMEM (Hyclone), supplemented with 10% fetal bovine serum (FBS, Hyclone), 100 U/ml penicillin and 100 U/ml streptomycin in a 37°C incubator with humidified atmosphere of 5% CO_2_ and 95% air.

### Preparation and identification of EMV

EMV were prepared from HBMEC under TNF-α and SD treatment (Wang et al., 2013). To generate EMV, HBMEC were cultured in SD medium supplemented with 10 ng/ml TNF-α (Sigma, St Louis, MO, USA) for 48 h. EMV were collected from HBMEC modified culture medium as previously described (Chen et al., 2011; Cantaluppi et al., 2012). In brief, the HBMEC culture medium was collected and centrifuged at 2000 g for 20 min to remove cells and debris. Then cell-free culture medium was ultra-centrifuged at 20,000 g for 90 min to pellet MV. The pellet MV were re-suspended with phosphate-buffered saline (PBS) filtered through 20 nm-filter (Whatman, Pittsburgh, PA), and aliquoted for nanoparticle tract analysis (NTA), transmission electron microscopy (TEM) and flow cytometry analysis.

EMV can be quantified by flow cytometry based on EC-related surface markers such as CD31, CD51, CD62E, and CD144 (Horstman et al., 2004; Amabile et al., 2005). To define EMV, samples were stained with 5 μL of PE-conjugated anti-mouse CD144 antibody (BD Biosciences). The size of vesicles was calibrated using 1 and 2 μm flow cytometry beads (Molecular Probes; Invitrogen, Eugene, OR). EMV were defined as vesicles with a diameter < 1 μm.

Morphology and size of sorted MV were further confirmed by TEM, quantified and averaged by examining four random microscopy fields (magnification, × 15,000).

### Co-culture assay of HBVSMC with EMV

In order to co-culture HBVSMC with EMV, the latter were labeled with PKH26 (Sigma Aldrich, St Louis, MO) according to the manufacturer's protocol with some modifications (Soleti et al., 2012). Briefly, the concentration of EMV was quantified by NTA, and 2 × 10^7^/mL EMV were used for co-culture experiments. EMV were labeled with 2 μM PKH26 (Sigma-Aldrich, St Louis, MO) at room temperature (RT) for 5 min. An equal volume of 1% bovine serum albumin (BSA) was added to stop staining. EMV were then ultra-centrifuged and re-suspended with culture medium. The PKH26-labeled EMV were added to HBVSMC seeded in glass plates for 24 h incubation (37°C, 5% CO_2_). Cell nuclei were then stained with DAPI (1 μg/mL; Wako Pure Chemical Industries Ltd). The merging of EMV by HBVSMC was examined under a fluorescence microscope (Leica, TCS SP5II, Germany).

### Gene expression analysis

The levels of miR-146a-5p in EMV, EMV treated with RNase A and HBVSMC were determined. Total miR were extracted by using miRNeasy Mini kit (QIAGEN) according to the manufacturer's instructions. The miR-146a-5p cDNA were synthesized using Hairpin-it™ miRNA RT-PCR Quantitation kit (GenePharma, Shanghai, China) using the following parameters: (25°C for 30 min, 42°C for 30 min, and 85°C for 5 min). Real-time PCR parameters were 95°C for 3 min; 40 cycles were performed at 95°C for 12 s and 60°C for 40 s. PCR primers were as follows: 5′-TGC CGC TGA GAA CTG AAT T-3′ and 5′-CAG AGC AGG GTC CGA GGT A-3′ for miR-146a-5p; 5′-CTC GCT TCG GCA GCA CA-3′ and 5′-AAC GCT TCA CGA ATTTGC GT-3′ for small nuclear RNA U6 (as an internal control). Quantitative real-time PCR was conducted on a real-time PCR system (Bio-Rad). Relative expression of miR-146a-5p was calculated using the 2^−ΔΔCT^ method (Cheng et al., 2013).

### Cell proliferation analysis

Cell proliferation of HBVSMC were tested using an MTT (3-[4,5-dimethylthiazyol-2yl]-2,5-diphenyltetrazolium bromide, 5 mg/mL, Sigma) assay. To eliminate the effects of RNA carried by EMV, the latter were pre-treated with 0.1% Triton-100 for 5 min, then treated with 200 U/mL RNase A (Thermo scientific, USA) for 90 min at 37°C then washed and pelleted by ultracentrifugation (Cantaluppi et al., 2012; Wang et al., 2013). To verify the effect of RNase, total RNA was extracted from EMV using the RNA isolation kit (Ambion, USA), and the RNA concentration was determined using quantitative assay (Biotek Epoch, Microplate reader, USA). The obtained RNase A-treated EMV were set as RNase-EMV. For proliferation analysis, cells were seeded into 96-well plates at a concentration of 2 × 10^3^ cells/well containing 200 μL of DMEM (supplemented with 10% FBS) containing EMV, RNase-EMV, or PBS (vehicle). The MTT solution (20 μL) was added and incubated with cells for 4 h at 37°C, then 150 μL of DMSO was added to each well and incubated with the cells for 20 min at 37°C. The optical density (OD) was read at 490 nm on a microplate reader (BioTek, USA). Measurement was carried out on day 3 after incubation. The percentage of cell proliferation was defined as the relative absorbance of treated cells versus untreated cells. Cells from 3 wells were counted at each time point, and the experiment was repeated 3 times for each group. Results are represented as the mean ± *SEM* from values obtained in 3 independent experiments.

### Cell migration assay

The migration rate of HBVSMC was measured by scratch assay (Yaghini et al., 2010). Cells were grown to confluence on 6-well cell culture plates. A scratch was made through the cell monolayer using a pipette tip. After washing with PBS, 0.5% FBS maintenance medium containing EMV, RNase-EMV, or PBS (vehicle) was added. Photographs of the wounded area were taken immediately after making the scratch (0 h time point) and 16 h after, to monitor the invasion of cells into the scratched area. For pathway blocking experiments, cells were pre-incubated with Mek1/2 inhibitor (PD0325901, 10 mM; Selleckchem) for 2 h. The experiment was repeated 3 times for each group.

### Apoptosis assay

Cell apoptosis was analyzed by Hoechst 33258 staining and Annexin V-PE/7-AAD apoptosis detection kit (BD Biosciences) as we previously described (Liu et al., 2015). Briefly, HBVSMC were seeded at a density of 2 × 10^5^/well in 2 mL serum free DMEM. Following co-culture with EMV, RNase-EMV, or PBS (vehicle) for 24 h, cell apoptosis was measured. Cells were fixed and stained with Hoechst 33258 solution according to the manufacturer's instructions (Beyotime) followed by fluorescence microscope observation in 5 independent fields assessed from each well. The average number of positive cells and total cells per field were determined. The apoptotic rate was defined as the ratio of positive cells versus total cells. For Annexin V-PE/7-AAD apoptosis detection, cells were washed with PBS, re-suspended with 100 μL 1X annexin-binding buffer, incubated with 5 μL PE-conjugated Annexin V and 5 μL 7-Aminno-actinomycin (7-AAD) for 15 min in the dark, then analyzed by flow cytometry. Cells stained with both Annexin V-PE and 7-AAD were considered to be late apoptotic HBVSMC, while cells stained only with Annexin V-PE were considered to be early apoptotic cells. Three plates per experiment were analyzed and the experiment was repeated 3 times for each group.

### Western blot

For western blot analysis, 30 μg protein of cells lysate were separated by 12% SDS-PAGE on Tris-glycine gels (Invitrogen) and transferred to PVDF membranes (Millipore Corp, Bedford, MA). The membranes were blocked 1 h at RT in TBS (50 mM Tris, 150 mM NaCl, pH 7.6, 5% fat-free dry milk) and washed in TBST (0.5% Tween20 in TBS), 2 min at RT. Primary antibodies were added overnight at 4°C then washed out in TBST. Secondary antibody (1:50,000, EarthOx, USA) was added for 1 h, at RT. Membranes were washed in TBST and detection was done using ECL solution (GE healthcare, USA). β-actin (1:1000, EarthOx, San Francisco, CA, USA) was used to normalize protein loading. The following primary antibodies were used: Mek1/2 (1:1000, CST, USA), Erk1/2 and phosphor-Erk1/2 (Thr202/Tyr204) (1:1000, CST, USA), Bcl-2 (1:1000, abcam, Britain), cleaved caspase-3 (1:1000, CST, USA).

### Statistic analysis

All data are expressed as mean ± *SEM*. Multiple comparisons were performed by two-way ANOVA. Comparisons for two groups were performed using a Student's *t*-test (GraphPad Prism 5 software). *p* < 0.05 were considered to be significant.

## Results

### Microvesicles characterization

**Figure 1:**
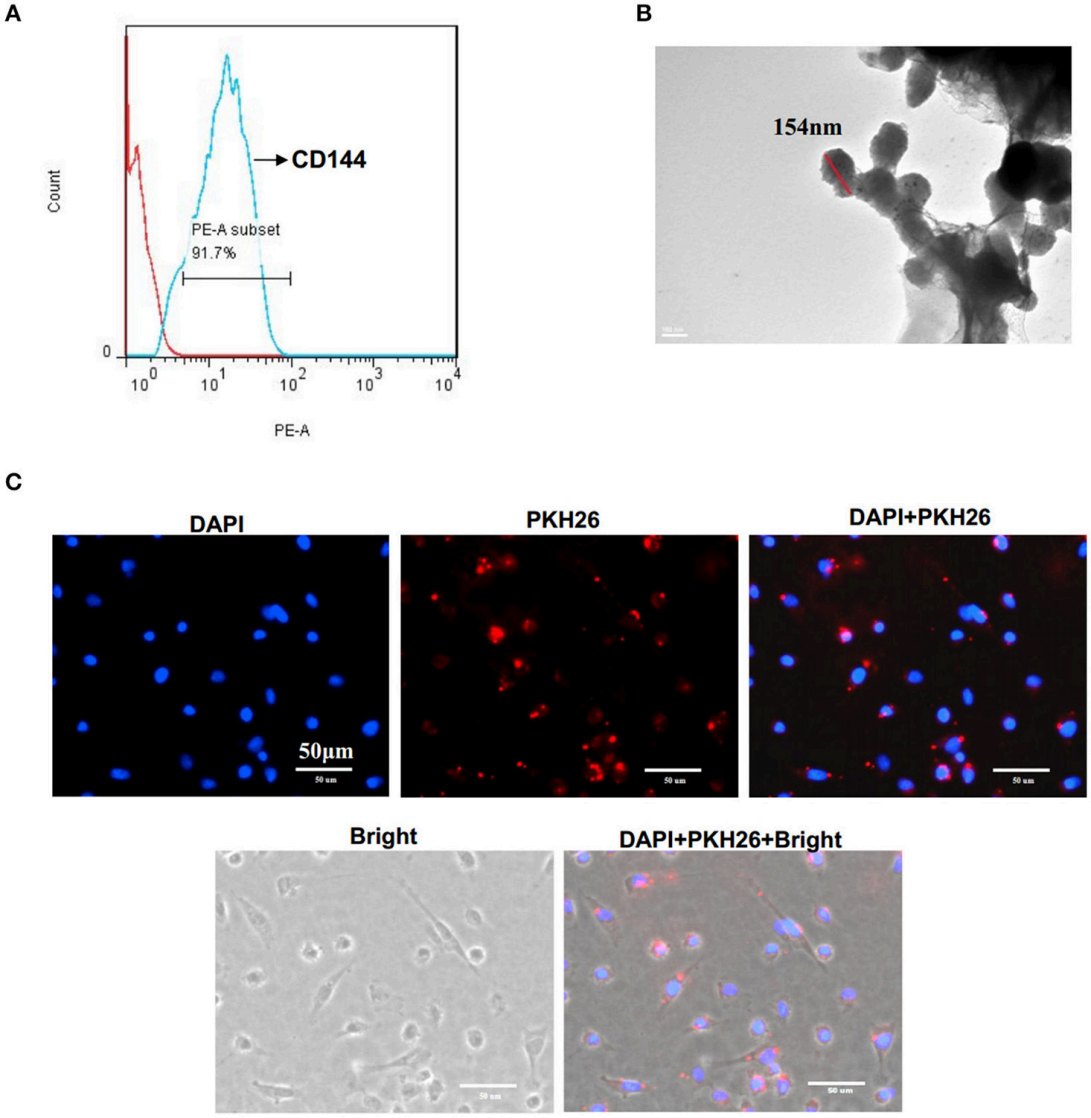
**Characterization of EMV. (A)** EMV were identified as 0.1–1.0 μm particles specifically stained with PE-CD144 by flow cytometry. **(B)** Representative image of EMV examined by TEM. **(C)** The incorporation of EMV with HBVSMC after co-culture. Representative images showing the merging of PKH26 labeled EMV with HBVSMC (red:PKH26;blue:DAPI). Scale bar: 50 μm.

EMV were identified as 0.1–1.0 μm nanoparticles specifically expressing the EC specific marker CD144. Flow cytometry analysis showed that EMV were 91.7 ± 1.1% positive for CD144 (Figure 1A). TEM analysis showed that the average size of EMV was 154 ± 14 nm in diameter (Figure 1B).

### EMV merged with HBVSMC after *In vitro* co-incubation

After co-incubation of PKH26-labeled EMV with HBVSMC for 24 h, PKH26 fluorescence was detected in the cytoplasm of HBVSMC (Figure 1C), suggesting that EMV merged with HBVSMC.

### RNase abolished the effect of EMV on increasing the proliferation of HBVSMC

**Figure 2:**
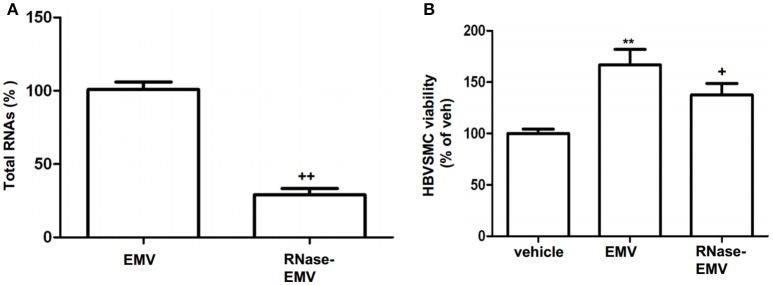
**RNase digestion of EMV and effects of EMV and RNase- EMV on viability of HBVSMC. (A)** Summarized data showing effective digestion of EMV total RNAs by RNase treatment. **(B)** Summary data showing that EMV promoted HBVSMC proliferation, and RNase-EMV was less effective. ^**^p < 0.01 vs. vehicle; ^+^*p* < 0.05,^++^*p* < 0.01 vs. EMV.

As shown in Figure 2A, following RNase digestion, total RNA in EMV was significantly decreased by 71.8 ± 2.8% (vs. EMV; *p* < 0.01; *n* = 3/group; Figure 2A). According to the MTT assay, we found that EMV increased the proliferation of HBVSMC by approximately 167% (vs. vehicle; *p* < 0.01; *n* = 3/group; Figure 2B). As expected, the effect of RNase-EMV on HBVSMC proliferation was reduced by approximately 30% compared to EMV (*p* < 0.05; *n* = 3/group; Figure 2B).

### RNase abolished the effect of EMV on increasing the migration ability in HBVSMC via Mek1/2/Erk1/2 pathway

**Figure 3:**
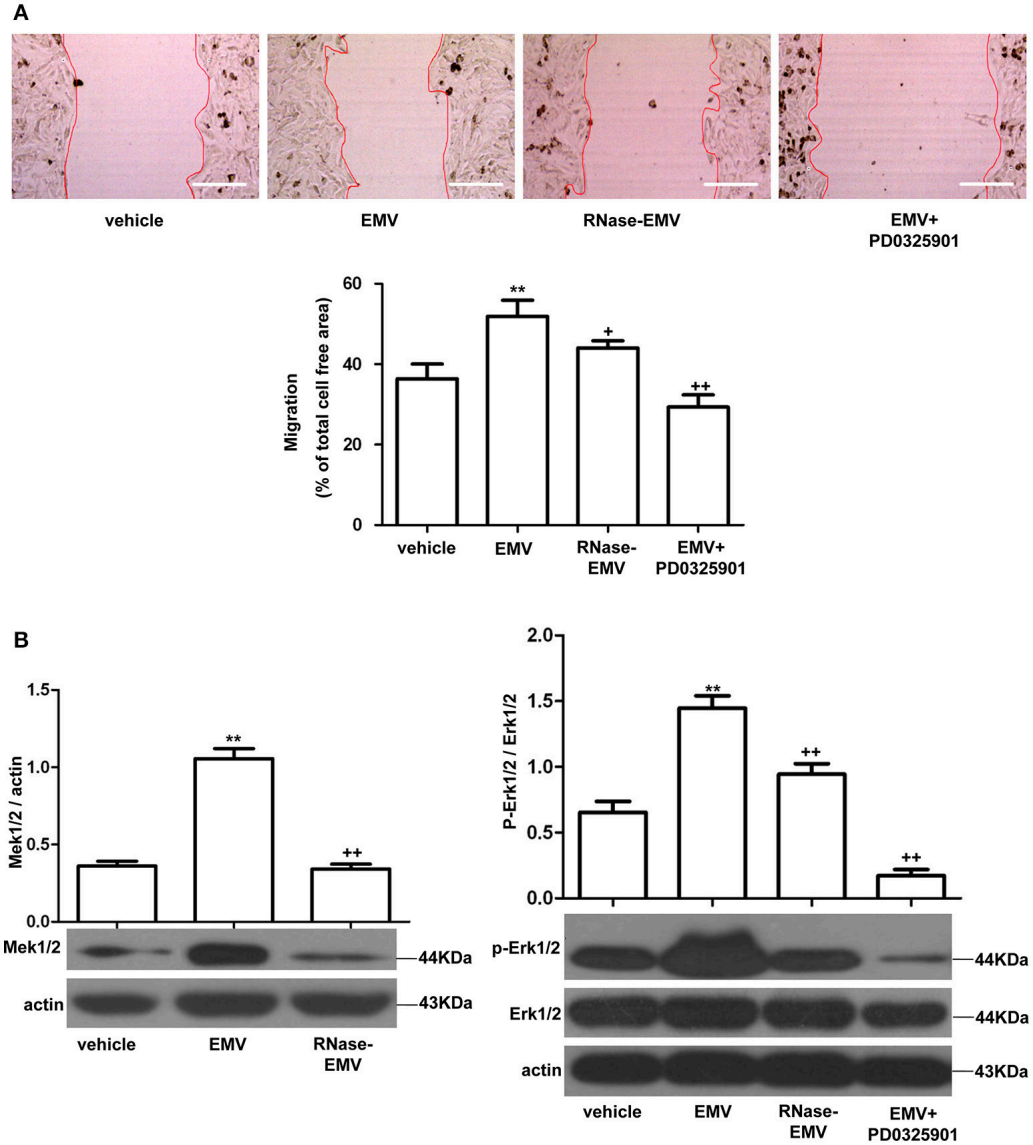
**Effects of EMV and RNase-EMV on migration and expression of Mek1/2 and p-Erk1/2/Erk1/2 in HBVSMC. (A)** Migration of HBVSMC treated with EMV, RNase-EMV or EMV+Mek1/2 inhibitor (PD0325901). **(B)** Expression of Mek1/2 and p-Erk1/2/Erk1/2. ^**^p < 0.01 vs. vehicle; ^+^*p* < 0.05, ^++^*p* < 0.01 vs. EMV. Scale bar: 400 μm.

The average migration area of HBVSMC was increased by 12.6 ± 2.3% in the EMV group (vs. vehicle; *p* < 0.01; *n* = 3/group; Figure 3A). RNase-EMV were less effective on increasing the migration ability of HBVSMC (vs. EMV; *p* < 0.05; *n* = 3/group). The data suggest that EMV promoted HBVSMC migration via their carried RNA. In addition, pre-incubation of HBVSMC with the Mek1/2 inhibitor PD0325901 also attenuated this effect (vs. EMV; *p* < 0.01; *n* = 3/group; Figure 3A), indicating that the Mek1/2 pathway contributed to the effects of EMV on HBVSMC migration.

EMV significantly up-regulated Mek1/2 protein expression and the phosphorylation of Erk1/2 in HBVSMC (vs. vehicle; *p* < 0.01; *n* = 3/group; Figure 3B), while RNase-EMV were less effective (vs. EMV; *p* < 0.05; *n* = 3/group). Pre-incubation of HBVSMC with PD0325901 also attenuated these effects (vs. EMV; *p* < 0.01; *n* = 3/group; Figure 3B). These data demonstrate that the migration promoting effect of EMV is closely related to the Mek1/2/ Erk1/2 pathway.

### RNase abolished the effect of EMV on reducing the apoptosis of HBVSMC accompanied with the change of caspase-3 and Bcl-2 level

**Figure 4:**
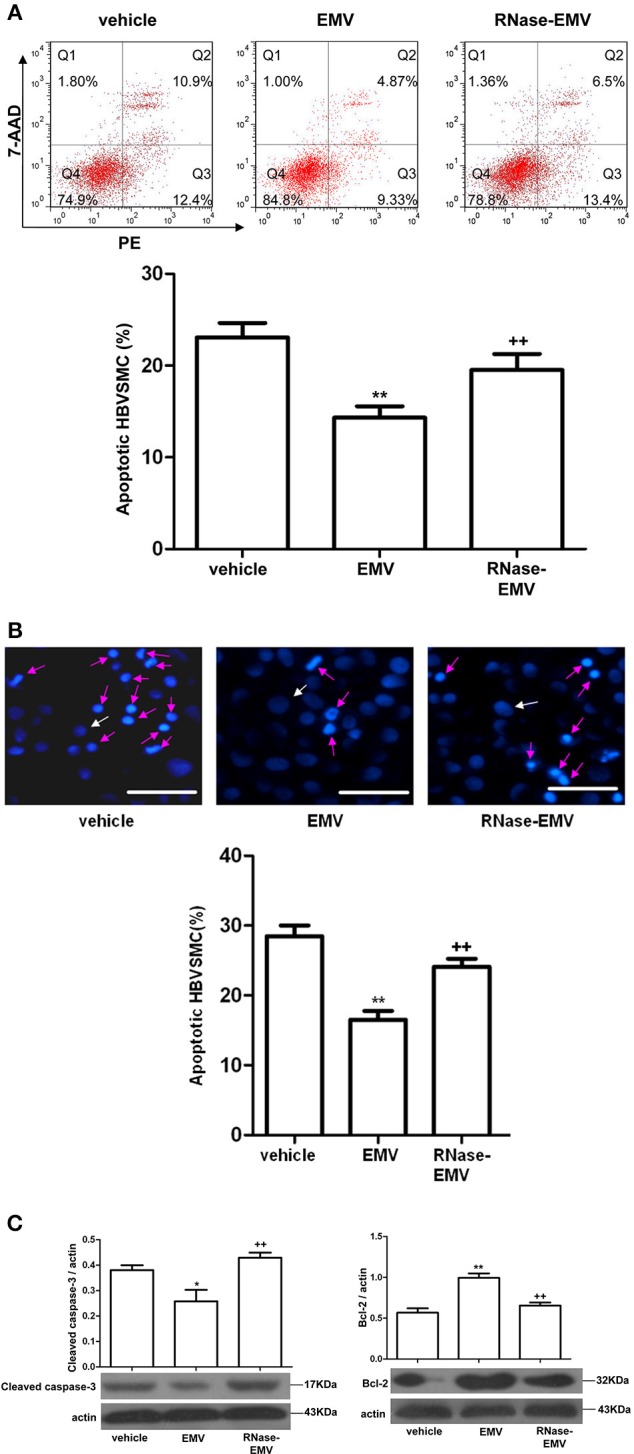
**Effects of EMV and RNase-EMV on apoptosis and expression of cleaved caspase-3 and Bcl-2 expression in HBVSMC. (A)** Apoptosis analysis by flow cytometry. **(B)** Apoptosis determined by Hoechst 33258 staining (Red arrows represent apoptotic cells, white arrows represent normal cells). Scale bar: 50 μm. **(C)** Protein levels of cleaved caspase-3 and Bcl-2 in HBVSMC. ^*^p < 0.05, ^**^p < 0.01 vs. vehicle; ^++^*p* < 0.01 vs. EMV.

Annexin V-PE/7-AAD and Hoechst 33258 staining revealed that EMV significantly decrease the apoptotic rate of HBVSMC by about 35% (vs. vehicle; *p* < 0.01; *n* = 3/group; Figures 4A,B). Following treatment by RNase, EMV exhibited a lower apoptotic inhibitory effect on HBVSMC, and the anti-apoptotic effect of RNase-EMV in HBVSMC decreased by nearly 70% (vs. EMV; *p* < 0.01; *n* = 3/group; Figures 4A,B).

In addition, we monitored the cleaved caspase-3 and Bcl-2 levels, which are associated with induction of apoptosis, by western blot. Results show that cleaved caspase-3 protein expression was significantly decreased (vs. vehicle; *p* < 0.05; *n* = 3/group; Figure 4C) while Bcl-2 protein expression obviously increased (vs. vehicle; *p* < 0.01; *n* = 3/group; Figure 4C) after EMV treatment. RNase-EMV also showed less effect (vs. EMV; *p* < 0.01; *n* = 3/group).

### RNase abolished the effect of EMV on increasing miR-146a-5p gene expression in HBVSMC

**Figure 5:**
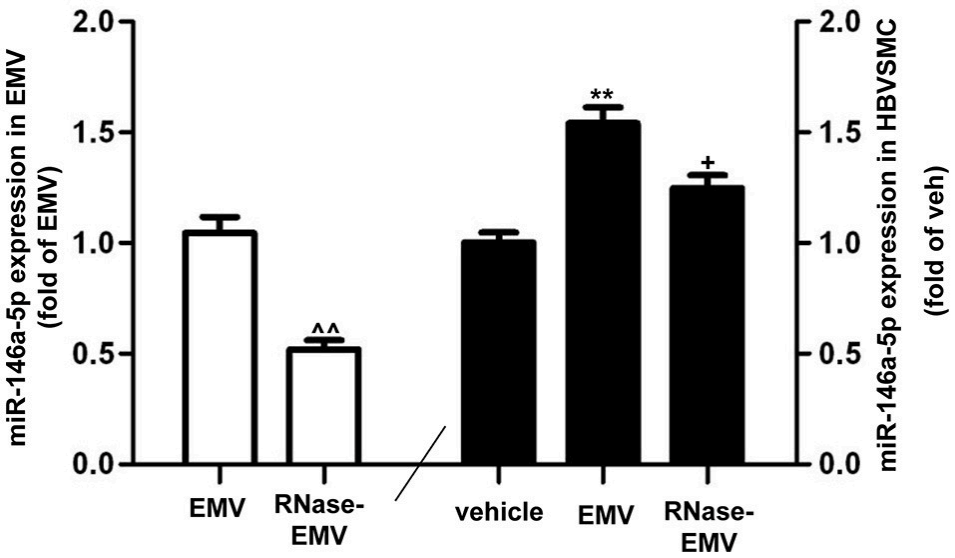
**MiR-146a-5p expression in EMV and HBVSMC**. Summary data showing effective digestion of miR-146a-5p in EMV by RNase treatment. ^∧∧^*p* < 0.01 vs. EMV. MiR-146a-5p expression in HBVSMC co-cultured with EMV or RNase-EMV was also measured. ^**^p < 0.01 vs. vehicle; ^+^*p* < 0.05, vs. EMV.

QRT-PCR data show that miR-146a-5p gene expression was significantly increased by nearly 2-folds in EMV-treated HBVSMC (vs. vehicle; *p* < 0.01; *n* = 3/group; Figure 5). The level of miR-146a-5p in RNase-EMV was significantly decreased by 48 ± 7% (vs. EMV; *p* < 0.01; *n* = 3/group, Figure 5). Meanwhile, the ability of RNase-EMV to increase miR-146a-5p expression in HBVSMC was impaired by nearly 20% (vs. EMV; *p* < 0.05; *n* = 3/group; Figure 5).

## Discussion

Accumulating evidence suggest that EMV could regulate the function of various cells, including EC, monocytes, dendritic cells, and T lymphocytes (Abid Hussein et al., 2007; Angelot et al., 2009; Lu et al., 2013). In this study, our results show that EMV secreted from HBMEC stressed with TNF-α plus SD could merge with HBVSMC and significantly increase cell proliferation and migration, while inhibiting cell apoptosis via their carried RNA. The effects of EMV on HBVSMC migration were associated with an increase in Mek1/2 and p-Erk1/2, which could be abolished by PD0325901, while the apoptosis inhibitory effect was accompanied with down-regulation of cleaved caspase-3 and up-regulation of Bcl-2. These results indicate that EMV mediate EC-VSMC communication, playing important roles in regulating vascular homeostasis. EMV released under an inflammatory environment can induce HBVSMC dysfunction, which may contribute to the pathogenesis of ischemia- and inflammation-related vascular diseases.

Inflammation contributes to the pathogenesis of various vascular diseases. TNF-α is a major inflammatory factor involved in the pathological basis of EC injury and AS (Dixon and Symmons, 2007; Zheng et al., 2013; Zhang et al., 2014). Previous studies have used SD to induce EC apoptosis, oxidative stress and dysfunction (Chen et al., 2010), which are known to contribute to vascular diseases (Kawashima and Yokoyama, 2004). EMV can deliver and transfer their contents (miRNA, mRNA, proteins) to target cells. Besides, EMV derived from different stimuli have shown different or even adverse effects on the recipient cells depending on their distinct contents (Wang et al., 2013; Pan et al., 2016; Paul et al., 2016). In this study, we generated EMV from HBMEC under TNF-α plus SD stimulation to mimic ischemia and inflammation in ischemic diseases, and examined the functional role of these EMV on HBVSMC. We observed that both proliferation and migration of HBVSMC were increased significantly after treated with EMV. It is well-known that VSMC proliferation and migration play a critical role in the pathogenesis of AS (Zheng et al., 2013). In the early phase of the disease, VSMC, changing from contractile to synthetic, migrate from media to the intima, and then proliferation of VSMC is thought to promote neointimal hyperplasia and remodeling (Geng and Libby, 2002; Sugimoto et al., 2010). Our findings suggest that MV derived from HBMEC under inflammation conditions such as TNF-α plus SD could contribute to the pathogenesis and progression of AS via amplification of VSMC migration and proliferation. Our earlier report has shown that MV released from endothelial progenitor cells (EPC-MV) treated with TNF-α and SD injured EC function, including increased cell apoptosis and ROS production, while decreasing NO cell production and tube formation (Wang et al., 2013). The detrimental effects of EPC-MV released under TNF-α plus SD environment are consistent with the findings on EMV of the present study. Additionally, we found that EMV significantly decrease HBVSMC apoptosis. It is well accepted that in the late stage of AS, activated inflammatory and immune cells in the plaque can lead to the death of VSMC by apoptosis, and finally cause plaque rupture and cerebral hemorrhage (Geng and Libby, 2002). Therefore, the inhibitory effects of EMV on HBVSMC apoptosis might also contribute to maintain the plaque stability. Thus, EMV may have complex functions in the process of AS. However, there are limitations in the present study. Our experiments were solely carried out *in vitro*. While we demonstrated that EMV could merge with BVSMC *in vitro*, this needs to be further determined in mice brain tissue. To confirm the function of EMV *in vivo*, primary cell cultures or *in vivo* treatments are warranted.

To determine whether the effects of EMV depend on the carried RNA, we treated EMV with RNase A as previously reported (Cantaluppi et al., 2012; Wang et al., 2013). We found that RNase A treatment diminished the effects of EMV on HBVSMC, confirming the hypothesis that EMV played roles via their carried RNA. Several microRNAs have been reported to participate in regulating VSMC function, such as miR-145/143 (Cheng et al., 2009), miR-221/222 (Choe et al., 2015), miR-146a (Sun et al., 2011), miR-34(Choe et al., 2015). A recent study reported that MV secreted by KLF2-transduced or shear-stress-stimulated human umbilical vein EC are enriched in miR-143/145 and could transfer these miRNA to co-cultured SMC, controlling target gene expression in these cells. These data show a communication between EC and SMC through a miRNA- and microvesicle-mediated mechanism. MiR-146a is well-known for its important regulatory role in the immune response and inflammation (Cheng et al., 2013). Recent studies have demonstrated that miR-146a promote VSMC proliferation and migration by targeting KLF4 (Sun et al., 2011). Herein, we found that EMV increased miR-146a-5p expression in HBVSMC and that miR-146a-5p level in RNase -EMV and HBVSMC co-cultured with RNase -EMV increased less, which was accompanied by diminished effects on HBVSMC. These data suggest that miR-146a-5p was delivered to HBVSMC from HBMEC (under inflammation challenge) via EMV and play a functional role in regulating HBVSMC proliferation and migration capabilities. Our results are in agreement with previous studies showing MV can deliver and transfer their contents (miRNA, mRNA) to target cells and regulate target cells function (Mause and Weber, 2010; Ratajczak and Ratajczak, 2016).

Of note, our results indicate that RNase partially abolished the effect of EMV on increasing the viability of HBVSMC. This could be explained by our observation that RNase partially decreased miR-146a-5p expression in EMV (Figure 5). In other words, the RNase was not able to fully digest EMV-carried RNA. Additionally, it is well-known that MV could modulate target cells function through transfer of their contents (RNA, protein, and DNA, Morel et al., 2004; Deregibus et al., 2007). Therefore, our data suggest that other EMV contents, such as protein and RNA, might also contribute to the regulation of HBVSMC function. We admit that the different effects of EMV on HBVSMC may involve various RNA and/or proteins. Further investigation usingmiR-146a-5p knock down or overexpression experiments is needed to verify the role of miR-146a-5p via a more mechanistic analysis. Moreover, we defined that RNase A significantly decreased the effects of EMV on HBVSMC, suggesting that distinct RNA carried by the EMV contribute significantly to the changes in EMV-treated HBVSMC. However, we did not determine whether the same RNA was able to trigger the various downstream signaling pathways involved in these changes. This indeed needs further work, such as digestion of RNAs in HBVSMC.

To further understand the regulatory mechanisms of EMV, we examined the Mek/Erk pathway which comprise important proteins related with the migration of VSMC (Jeong et al., 2015; Lin et al., 2016). In this study, we found that EMV increased Mek1/2 and p-Erk1/2 in HBVSMC and blockade of the Mek1/2/Erk1/2 pathway inhibited the effect of EMV on promoting migration of HBVSMC. The results suggest that EMV promote migration of HBVSMC via activation of the Mek1/2/Erk1/2 pathway, thereby providing a probable underlying mechanism in the process of pro-atherosclerosis. The Mek-Erk pathway has been shown to be involved in VSMC proliferation and migration under an inflammatory environment (Lin et al., 2016). Nevertheless, whether it plays a role in VSMC apoptosis has not been investigated. Caspase-3 is an important apoptosis-promoting factor, which plays a critical role in the execution-phase of cell apoptosis (Kluck et al., 1997). Whereas, Bcl-2 is an anti-apoptotic protein, which serve as a key regulator at the early stage of apoptosis (Kluck et al., 1997). A recent study reported that up-regulation of Bcl-2 and inactivation of caspase-3 involved in the anti-apoptosis effect of Niacin on VSMC (Su et al., 2015). Herein, our data show that the EMV-mediated reduction of apoptosis of HBVSMC appears linked with Bcl-2 and cleaved caspase-3 signaling pathways. In addition, MV can deliver and transfer their contents (miRNA, mRNA, proteins) to target cells and regulate target cells functions. In this study, we explored the mechanisms underlying the effects of EMV on HBVSMC apoptosis by measuring the expression of apoptosis-related genes capase3 and Bcl-2. However, we only determined the level of miR-146a-5p in EMV, which has been shown to promote VSMC proliferation and migration (Wang et al., 2015). Our results show that the level of miR-146a-5p in HBVSMC was increased after EMV treatment, accompanied by an increase of Mek1/2/Erk1/2 expression. Meanwhile, after RNase digestion of EMV, miR-146a-5p level was decreased in EMV and EMV-treated HBVSMC, and the expression of Mek1/2/Erk1/2 in HBVSMC was also reduced. These findings indicate that miR-146a-5p is engaged in the expression of Mek1/2/ErK1/2. Future studies will focus on the contents of EMV to better understand the mechanisms underpinning the protective effect of EMV on caspase-3 and Bcl-2 expression.

## Conclusions

In conclusion, our data demonstrate that MV derived from HBMEC under inflammatory stimulation could significantly increase proliferation and migration of HBVSMC while reduced apoptosis of HBVSMC via their carried RNA is associated with the Mek1/2/Erk1/2 and caspase-3/Bcl-2 pathways, which might contribute to the pathogenesis of AS. Moreover, EMV mediate EC-SMC communication which could provide novel therapeutic targets for vascular diseases.

## Authors contributions

QP, HL, CZ, YZ, XL, YW, XM performed experiments; QP, XM, YY wrote the manuscript; QP, HL, CZ, YC, BZ, EL, YY, XM contributed to manuscript preparation; All authors read and approved the final version of this manuscript for submission.

## Funding

This work was supported by National Natural Science Foundation of China (NSFC, no. 81400360, 81270195); Science and Technology Planning Project of Guangdong Province (no. 2014A020212293); Medical Scientific Research Foundation of Guangdong Province (no. B2014311, A2016479, A2016485).

### Conflict of interest statement

The authors declare that the research was conducted in the absence of any commercial or financial relationships that could be construed as a potential conflict of interest.
